# bFGF-Regulating MAPKs Are Involved in High Glucose-Mediated ROS Production and Delay of Vascular Endothelial Cell Migration

**DOI:** 10.1371/journal.pone.0144495

**Published:** 2015-12-07

**Authors:** Zhong Xin Zhu, Wan Hui Cai, Tao Wang, Hong Bo Ye, Yu Ting Zhu, Li Sha Chi, Yuan Meng Duan, Cong Cong Sun, Yuan Hu Xuan, Li Tai Jin

**Affiliations:** 1 School of Pharmaceutical Sciences, Key Laboratory of Biotechnology Pharmaceutical Engineering, Wenzhou Medical University, Wenzhou, 325000, China; 2 Department of Biology, Stanford University, Stanford, 94305–5020, United States of America; Massachusetts General Hospital/Harvard Medical School, UNITED STATES

## Abstract

High blood sugar is a symptom of diabetes mellitus (DM). Vascular endothelial cells (VECs) directly contact the blood and are damaged when blood sugar levels are high. However, the molecular mechanism underlying this process remains elusive. To analyze the effects of DM on migration, we simulated DM by applying high glucose (HG) to the human VEC. HG delayed cell migration and induced phosphorylation of MAPKs (JNK and ERK). By contrast, in presence of bFGF, cell migration was promoted and MAPK phosphorylation levels were reduced. Furthermore, treatment with JNK and ERK inhibitors rescued HG-mediated delay of cell migration. Molecular and cell biological studies demonstrated that HG increased ROS production, whereas treatment with bFGF or JNK/ERK inhibitors blocked HG-induced ROS accumulation. Addition of MnTMPyP, a ROS scavenger, reduced HG-induced ROS production and accelerated cell migration, suggesting that the influence of HG on bFGF–MAPK signaling causes accumulation of ROS, which in turn regulate cell migration. This is the first study to elucidate the molecular mechanism of HG-mediated VEC migration; these findings could facilitate the development of novel therapies for DM.

## Introduction

Diabetes mellitus (DM) is one of the most intractable chronic diseases, affecting large numbers of people worldwide. The vascular complications caused by high blood glucose increase the probabilities of morbidity and mortality in diabetic patients. Early in the course of diabetes, intracellular hyperglycemia is associated with endothelial dysfunction and reduces neovascularization [1–3]. With time, endothelial cell (EC) loss by apoptosis and led to microvascular rarefaction, which limits the benefit of revascularization [4]. The overall consequence is tissue dysfunction, which results in the formation of non-healing ulcers.

Wound repair in the skin is a coordinated process that involves cell proliferation, cell migration, wound contraction, vasculogenesis, collagen deposition and remodeling. Various cell types participate in wound repair, primarily keratinocytes, fibroblasts, and vascular endothelial cells [5, 6]. Because of their inability to regulate glucose influx, VECs represent an important target for DM-induced damage. Extensive studies have demonstrated that diabetic conditions alter cell migration. A recent study showed that fibroblasts of diabetic mice migrated more slowly than those of mice with normal blood sugar [7]. This inhibitory effect on migration was also observed in keratinocytes incubated in a HG environment [8], indicating that HG plays a crucial role in cell migration. However, these studies did not reveal the cellular regulatory mechanisms involved in this phenomenon.

Wound healing processes are also regulated by numerous growth factors. bFGF is a member of the FGF protein family, which modulates the growth, differentiation, migration, and survival of a wide variety of cell types, including endothelial cells [9]. bFGF binds to the extracellular region of the FGF receptor (FGFR1) to regulate downstream components, such as MAPK family proteins, which play important roles in cell migration. In addition, bFGF activates the PI3K–Rac1–JNK pathway, which promotes fibroblast cell migration [10]. A recent study of the effects of HG on different cell types revealed that HG-induced oxidative stress abnormally activates Rac1, a small GTPase, thereby delaying cell migration [11]. bFGF promotes skin regeneration in diabetic rats [12], and VECs are among the major targets of bFGF in wound healing, but the relationship between DM and bFGF in VECs has not been thoroughly characterized.

In this study, we investigated diabetes-mediated impairment of blood vessel formation in type 1 diabetic rat and HG-induced inhibition of cell migration by analyzing the effects of HG on several migration-related parameters in VECs. HG stress activated reactive oxygen species (ROS) production and slowed cell migration. Furthermore, we identified ERK and JNK, two key MAPK family proteins involved in cell migration, as the mediators of HG-induced ROS production and delayed cell migration. Therefore, it is reasonable to conclude that HG activates ERK and JNK. This phenomenon was inhibited by activation of bFGF signaling. Consistent with this, bFGF reverted most of the migratory effects caused by HG. Taken together, our data indicate that hyperglycemia impairs cell migration by increasing ROS production, which was associated with activation of MAPK family proteins.

## Material and Methods

### 1. Cell culture and high-glucose experiments

EA.hy926 cells, a human endothelial cell line, were obtained from the Cell Bank of the Chinese Academy of Sciences (Shanghai, China). This cell line was immediately expanded and frozen so that they could be restarted every 2 to 3 months from a frozen vial of the same batch of cells. Cells were cultured in low-glucose (LG) DMEM supplemented with 10% fetal bovine serum and 1% penicillin–streptomycin. The cultures were maintained at 37°C in a humidified atmosphere (5% CO_2_ and 95% air), and the medium was refreshed every 2 days. Cultured cells were digested and passaged with 0.25% trypsin (Gibco) after reaching a confluence of approximately 90%. Trypsinized cells were seeded at 5 × 10^5^ cells /well on 6-well plates, at 5 × 10^4^ cells/well on 48-well plates, or at 5 × 10^3^ cells/well on 72-well plates, and cultured for 24 hours. The cells were then treated with 5.5 mM or 35 mM glucose for 3 days.

### 2. Cell proliferation assay

Cell proliferation was assayed as previously described [13]. In brief, cell resuspension solution containing approximately 2 × 10^3^ cells was placed into 96-well plates, and five duplicate wells were used for each treatment condition. After cell attachment, the media were replaced as indicated. Subsequently, 10 μL of CCK-8 (Dojindo Bio., Japan) was added to each well, and the cells were cultured for another 3 hours. Cell density was determined by measuring absorbance at 450 nm using a Varioskan Flash multimode reader (Thermo Scientific, Waltham, MA, USA). To analyze the effects of HG and bFGF on proliferation, 3000 cells were plated in 96-well plates. The medium was then inoculated with various concentrations of glucose, and the cells were incubated for 3 days before addition of the indicated concentrations of bFGF. Finally, CCK-8 was added, and OD was measured.

### 3. Wound scratch assay

Cell migration was determined using the wound scratch assay as described previously [10]. 4 × 10^4^ cells were seeded on a 3.5 cm dish, cultured for 3 days in LG or HG media, and then transferred to medium containing low FBS (0.5%) and 5 mg/mL mitomycin C. The cells were pretreated with LG or HG for 72 hours and then incubated with 100 ng/mL bFGF, 0.5 μM or 1 μM p-JNK inhibitor sp600125 (Millipore), 0.2 μM or 0.5 μM p-ERK inhibitor U0126 (Cell signaling technology), 10 μM or 20 μM ROS scavenger MnTMPyP (CALBIOCHEM) for 1 hour before wounding. Images of the wounded cell monolayers were acquired using a microscope (model IX70; Olympus, Tokyo, Japan) equipped with a CCD camera (CoolSNAP HQ; Nippon Roper, Chiba, Japan) at 0, 12, and 24 hours after wounding. The healing rate was quantified using measurements of the gap size after the culture. Ten different areas in each assay were chosen to measure the distance of migrating cells to the origin of the wound edge.

### 4. Measurement of intracellular reactive oxygen species (ROS)

VECs were treated with HG for 72 hours, and then treated with bFGF (100 ng/mL), sp600125 (1 μM), U0126 (1 μM), or MnTmMPyP (1 μM) for 1 hour. Intracellular ROS levels were estimated by addition of 10 mM 2’,7’-dichlorofluorescein diacetate (DCFH-DA) [13] to the medium; this compound is converted to dichlorofluorescein (DCF) in proportion to ROS concentration and remains trapped inside the cell. Before fluorescence analysis, cells were incubated for 30 minutes at 37°C in the dark and washed three times with 1X PBS, and then images of intracellular DCF (representing ROS levels) were obtained by fluorescence microscopy (Nikon, Japan, Tokyo) with excitation at 488 nm and emission at 610 nm. ROS levels were digitally quantitated using the Image Pro Plus software, version 6.0 (Media Cybernetics, USA).

### 5. Western blot analysis

Cells were lysed in ice-cold lysis solution (7 M urea, 2 M thiourea, 2% CHAPS, 40 mM Trizma base, 40 mM dithiothreitol [DTT], 1% protease inhibitor). After centrifugation at 15,000 × *g* for 15 min, total protein concentration in the supernatant was measured using the BCA protein assay (Pierce). Equal amounts of protein were loaded and separated on SDS-polyacrylamide gels, and then transferred onto PVDF membranes (Bio-Rad). The membranes were incubated for 1 hour in TBS containing 5% skim milk or 5% BSA and 0.05% Tween-20, and then incubated overnight with primary antibodies at 4°C. Primary antibodies were anti-cleaved Caspase-3 (1:1000, Cell Signaling Technology, 9661), anti-phospho-SAPK/JNK (Thr183/Tyr185) (1:1000, Cell Signaling Technology, 4668), anti-SAPK/JNK (1:1000, Cell Signaling Technology, 9252), anti-phospho-p44/42 MAPK (ERK1/2) (Thr202/Tyr204) (1:1000, Cell Signaling Technology, 9101), anti-p44/42 MAPK (ERK1/2) (1:1000, Cell Signaling Technology, 9102), anti-β-actin antibody (1:500, Santa Cruz Biotechnology, 47778), and anti-TNF-alpha (1:2000, Abcam, Cambridge, USA, ab1793). Membranes were then incubated for 90 min with HRP-conjugated anti-mouse or anti-rabbit secondary antibody (1:2000, Cell Signaling Technology). Finally, after washing, antigen-antibody complexes were visualized using an electrochemiluminescence (ECL) kit (GE Healthcare). Quantitation of relative band intensities was performed by scanning densitometry using the ImageJ software.

### 6. Immunofluorescence staining and fluorescence microscopy

Immunofluorescence staining was performed using the assay as described previously [14]. Briefly, fixed samples from each group were washed with PBS and permeabilized with 0.1% Triton X-100. After blocked with 0.5% BSA, samples were incubated with the primary antibody: anti-active Caspase-3 antibody (1:200, Abcam, Cambridge, USA, ab32042). Samples were incubated with corresponding Alexa Fluor-488 secondary antibody and the nuclear stain DAPI. Cellular images were examined with a fluorescence microscope (Nikon, Japan, Tokyo). All images were captured at the same exposure time, and presented values were corrected for local background fluorescence. Fluorescence intensity was measured with the Image Pro Plus software, version 6.0 (Media Cybernetics, USA).

### 7. Creation of wounds and application of bFGF

All animals were from the Laboratory Animals Center of Wenzhou Medical University and all procedures were carried out in accordance with the international ethical guidelines and the National Institutes of Health’s Guide for the Care and Use of Laboratory Animals. Male SD rats were made diabetic by injection of 60 mg/kg STZ dissolved in the sodium citrate buffer (pH 4.5) as described previously [12]. Age matched rats serving as control were given the same volume of sodium citrate. Only rats showing consistently elevated fasting glucose levels (16.7 mM) were included in the study [15]. Rats were anesthetized with pentobarbital (45 mg/kg) to create two full-thickness circular wounds (about 250 mm^2^ each) in the dorsal region by means of a pair of sharp scissors. 30 rats were divided into 3 groups: Normal (skin-wounded non-diabetic rats, n = 10), DM (skin-wounded diabetic rats, n = 10) and DM+bFGF (skin-wounded diabetic rats treated with bFGF, n = 10). bFGF (100 ng/mL, dissolved in saline) was applied to the wounded area of the diabetic group (0.5 mL for each area) every other day, while the equal amounts of saline were treated to normal and DM groups for 7 days [16].

### 8. Histopathological assay

Histopathological assay for blood vessel formation was obtained as described elsewhere [17]. Briefly, skin specimens were obtained from each group on day 7 after wounding for histopathological examination. Specimens were immediately fixed in 10% (v/v) neutral buffered formalin, and the solution was replaced every 2 days until the tissues had hardened. Each specimen was embedded in a paraffin block and 20 thin sections in one specimen (3 μm) were prepared and stained with haematoxylin and eosin (H&E) for blood vessel formation. The vessel numbers in each group representing the blood vessel density were evaluated by 2 blinded observers.

### 9. Statistical analysis

Statistical calculations were performed using Prism 5 (GraphPad, San Diego, CA, USA). All data are expressed as the means ± SE. For experiments involving > 2 groups, we used ANOVA with Turkey multiple pairwise comparisons. The threshold for statistical significance was *p* < 0.05.

## Results

### 1. bFGF treatment protects DM-mediated impairment of blood vessel formation in rats

**Fig 1:**
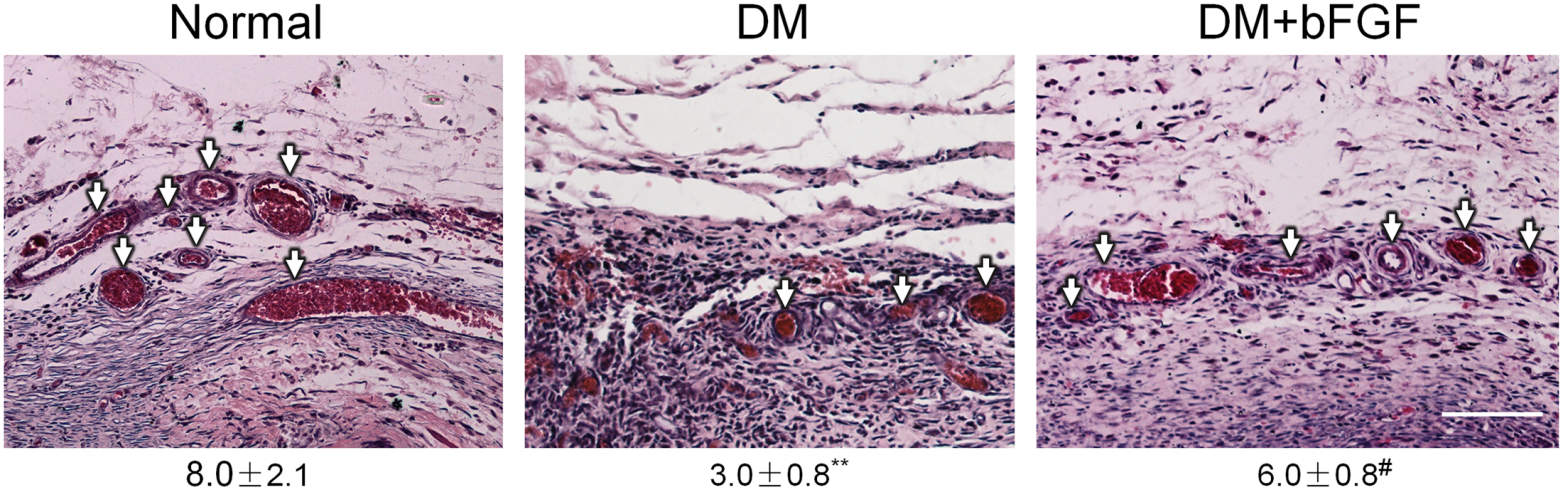
HE staining for blood vessel formation in rat skin at day 7 after treatment with bFGF. Normal: non-diabetic rat. DM: diabetic rat. DM+bFGF: diabetic rat with bFGF addition. White arrows indicate the blood vessels. The number shown in the bottom of each image represents vessel numbers, mean values ± SE of ten independent slides. Significant differences between the normal and DM group or DM and bFGF treated DM group are shown (**P < 0.01, ^#^ *P* < 0.05). Bars = 100 μm.

Full-thickness wound healing involves migration of keratinocytes, fibroblasts and endothelial cells to the wound bed prepared with appropriate ECM molecule deposition and subsequent neovascularization [18]. Neovascularization is a crucial step in the wound healing process [19]. When tissues are wounded, damaged blood vessels recruit, endothelial cells also participate in the wound repair process through enhancement of proliferation, migration, and angiogenesis [20]. A type 1 diabetic rat model was established to analyze the blood vessel formation. Eight weeks after the STZ injection, two full thickness circular wounds were created on the waist of each rat. Fig 1 shows the angiogenesis of each experimental group at day 7 after treatment. The data indicated that DM inhibited vessel density are lower than in non-diabetic and bFGF treated groups.

### 2. bFGF alleviates HG-induced reductions in VEC viability and migration

**Fig 2:**
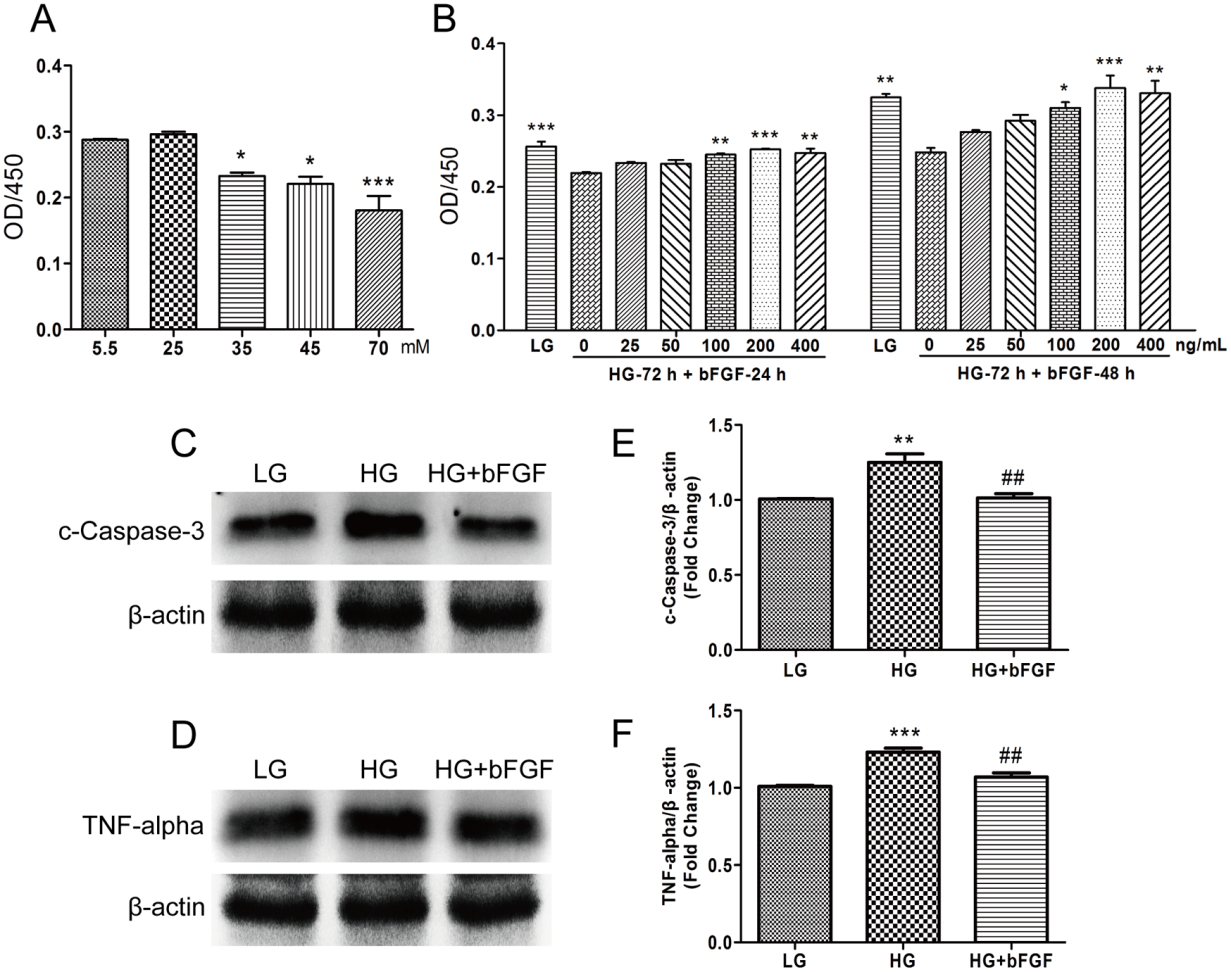
bFGF protects VECs against the deleterious effects of HG on VEC viability and proliferation. (A) Cell proliferation after 72 hours of HG treatment (25–70 mM), and (B) proliferation of cells treated with different concentrations of bFGF for 24 or 48 hours following 72 hours of HG treatment, measured by CCK-8 assay. (A) and (B) data represent mean values ± SE of four independent experiments (*P < 0.05, **P < 0.01, ***P < 0.001). Immunoblotting analysis for detection of cleaved Caspase-3 (C) and TNF-α (D) in VECs treated with HG (35 mM) for 72 hours, and then incubated with 100 ng/mL bFGF for 1 hour. LG: 5.5 mM glucose in culture medium. Signal intensities for cleaved Caspase-3 (E) and TNF-α (F) were normalized to that of β-actin. Results are presented as fold changes relative to VECs grown in medium containing 5.5 mM glucose (LG). (E) and (F) Data represent mean values ± SE of four independent experiments, relative to the LG group (**P < 0.01, ***P < 0.001) and the HG group (##*P* < 0.01).

**Fig 3:**
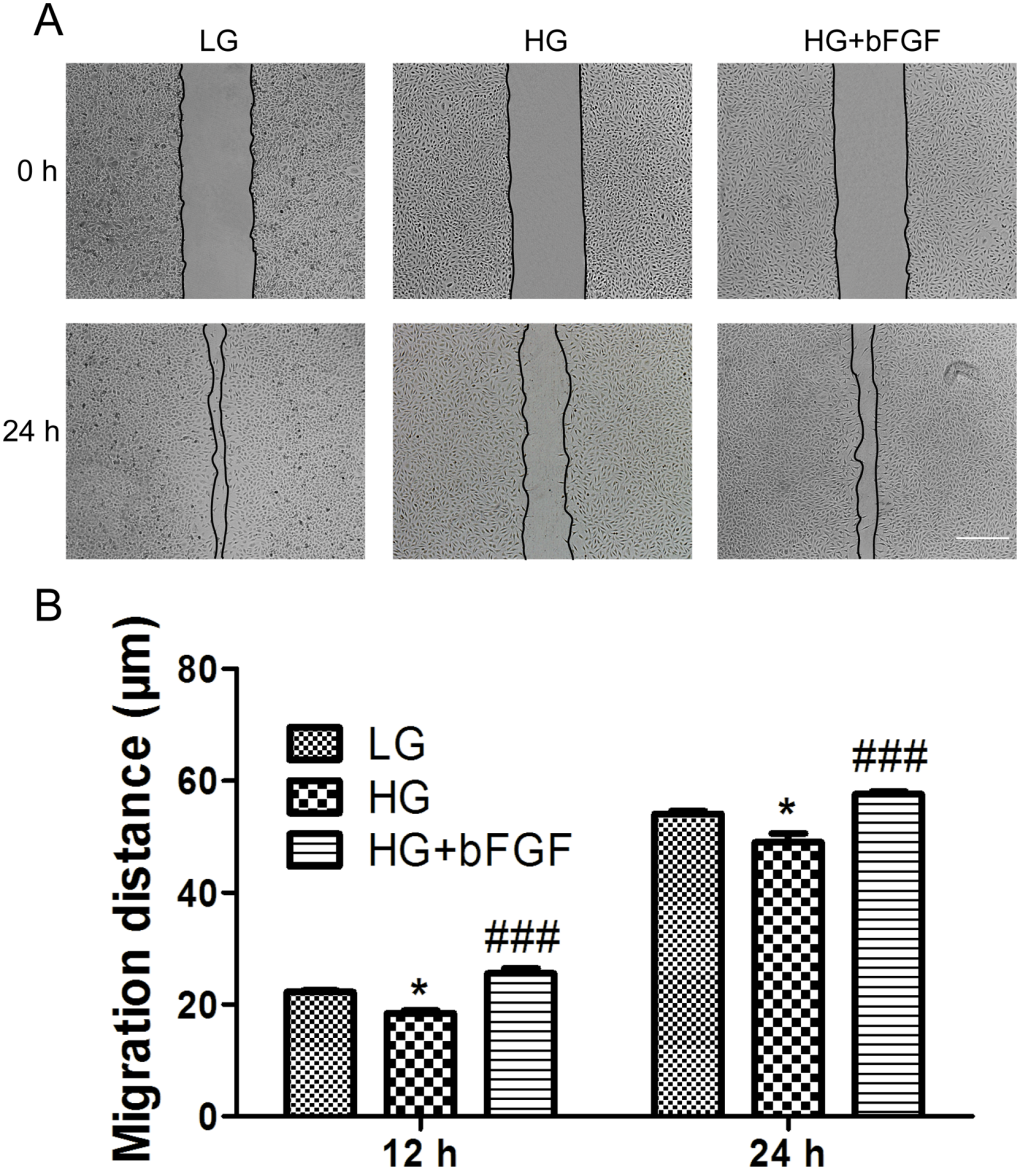
Effects of HG and bFGF on VEC migration. (A) Wound scratch assays were performed to analyze the effects of HG and bFGF on VECs. LG: medium containing 5.5 mM glucose. Bar = 500 μm. (B) Cell migration distance was determined according to the data shown in (A). Data represent mean values ± SE of ten replicates, relative to the HG group (*P < 0.05, ***P < 0.001). All experiments were performed after cells were treated with 5 mg/mL mitomycin C (to inhibit proliferation) for 1 day.

Endothelial cell migration is essential to angiogenesis [21]. To analyze the effects of high blood sugar on human VECs, which were required for new vessel formation, we added high concentrations of glucose to the culture medium to mimic a diabetes-induced high blood sugar environment. Cells were initially maintained in medium containing 1 g/L (5.5 mM) of glucose (low glucose, LG). For high glucose (HG) conditions, cells were transferred to media containing 25–70 mM glucose for 72 hours, and the effects of HG on wound healing, including cell proliferation and migration, were monitored (Figs 2A and 3A). HG concentration at 35 mM was relevant to diabetic patients with severe hyperglycemia and has been used in many prior studies of the vascular effects of high glucose concentrations. HG obviously inhibited proliferation and migration relative to the LG condition. bFGF modulates the growth, differentiation, migration, and survival of multiple cell types [22], and a recent study showed that bFGF can even reverse HG-induced apoptosis and inflammation in fibroblasts [13]. Therefore, we investigated whether bFGF could protect cells against HG (35 mM)-induced damage. To test the effects of bFGF on proliferation and migration, we treated VECs with different concentrations of bFGF (25–400 ng/mL). Cells were counted 48 or 72 hours after addition of bFGF (Fig 2B). Wound scratch assays were performed to examine the effects of bFGF on the HG-induced delay in cell migration (Fig 3A and 3B). To exclude interference from cell proliferation, we pre-treated the cells for 1 day with the mitosis inhibitor mitomycin C; preliminary experiments revealed that 5 mg/mL was the optimal concentration (data not shown). bFGF promoted cell proliferation and migration of HG-stressed VECs and suppressed HG-mediated induction of apoptosis and inflammation marker proteins (cleaved Caspase-3 and TNF-alpha) (Fig 2C, 2D, 2E and 2F). These results suggested that HG triggered cellular damage in VECs, whereas bFGF reversed the HG-induced reductions in viability and migration.

### 3. HG suppresses cell migration via activation of MAPKs (JNK and ERK)

**Fig 4:**
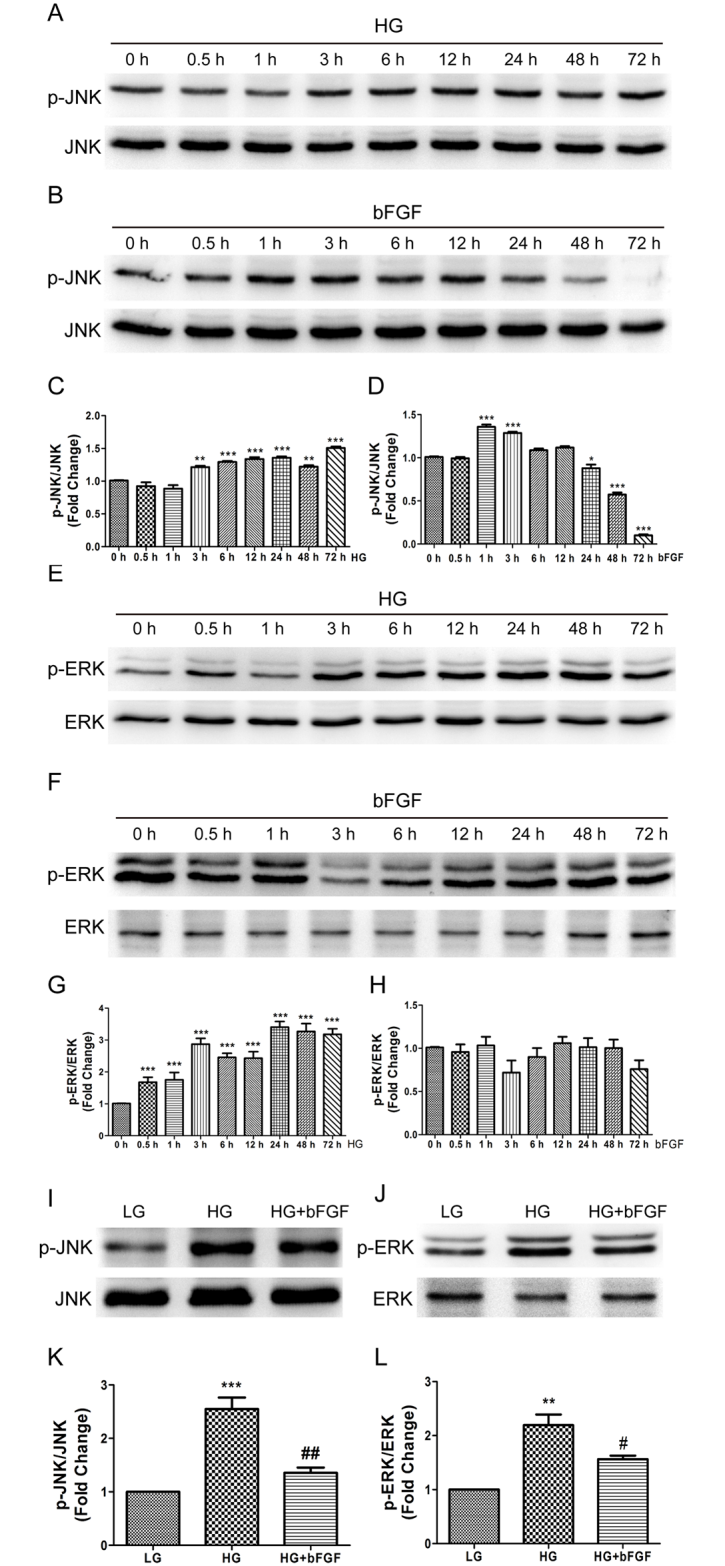
Effects of HG and bFGF on phosphorylation of JNK and ERK. Immunoblotting analysis was performed to analyze the effects of high glucose (HG) and 100 ng/mL bFGF on phosphorylation levels of JNK and ERK respectively (A, B, E, F) during 72 hours of period and effects of 30 minutes of bFGF stimulation before HGstressed human VECs for 72 hours. All experiments were performed after exposure of cells to 5 mg/mL mitomycin C (to inhibit proliferation) for 1 day. (C), (D), (G), (H), (K), (L) Results are presented as fold changes relative to LG-incubated VECs. Data represent mean values ± SE of four independent experiments, relative to the LG group (*P < 0.05) and the HG group (^#^ *P* < 0.05, ^##^ *P* < 0.01).

**Fig 5:**
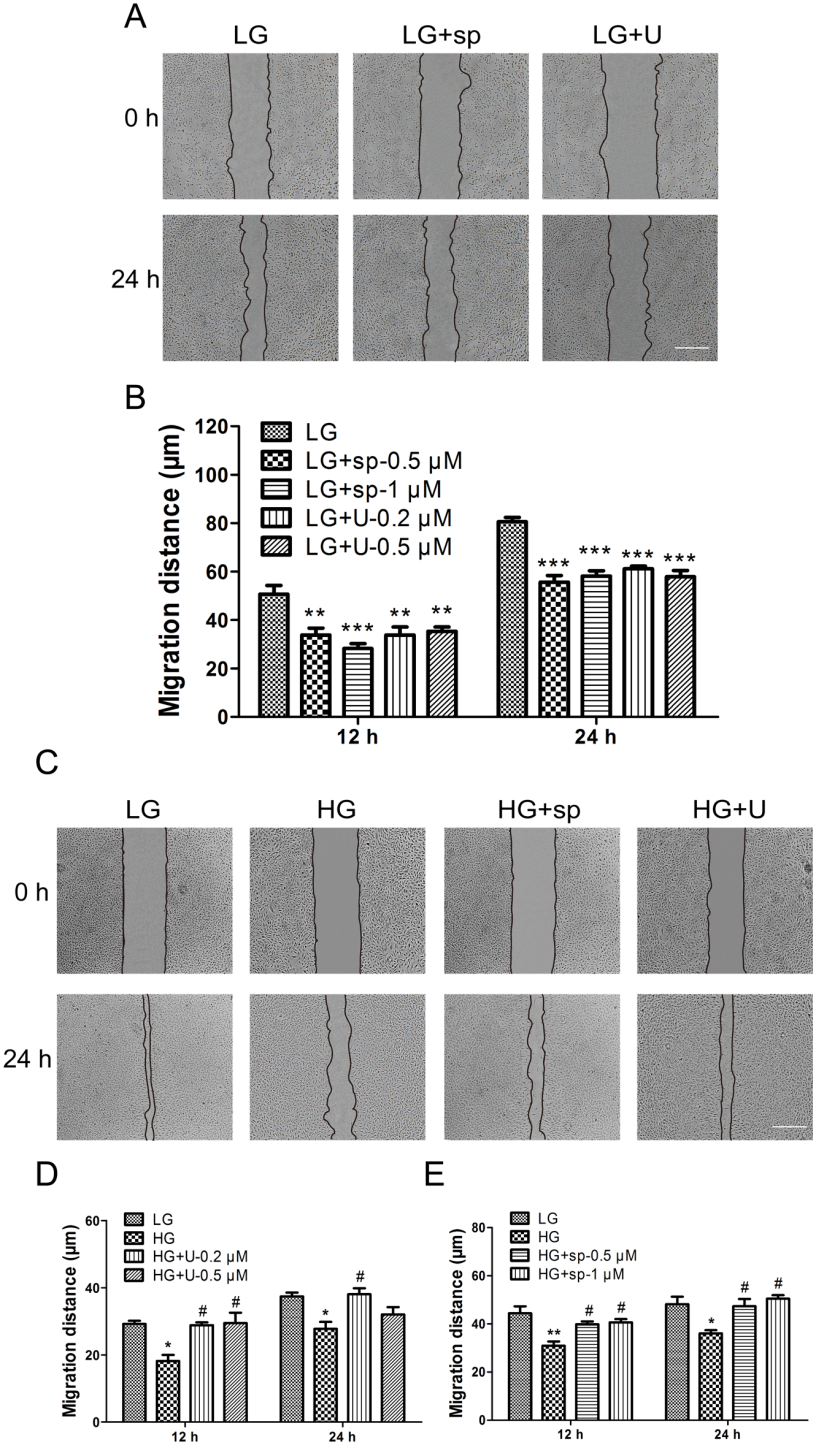
Effects of HG and JNK and ERK inhibitors on VEC migration. (A), (C) Wound scratch assay was performed to analyze the effects of high glucose (HG), the JNK inhibitor sp600125 (sp), and the ERK inhibitor U0126 (U) on human VECs. LG: medium containing 5.5 mM glucose; HG: medium containing 35 mM glucose. The concentration of sp600125 was 1 μM, and the concentration of U0126 was 0.2 μM. Bar = 500 μm. (B), (D), (E) Cell migration distance was determined according to the data shown in (A) and (C). All experiments were performed after exposure of cells to 5 mg/mL mitomycin C (to inhibit proliferation) for 1 day. Data represent mean values ± SE of ten replicates, relative to the LG group (*P < 0.05, **P < 0.01, ***P < 0.001) and the HG group (*P < 0.05, **P < 0.01).

bFGF activates multiple downstream signals, including mitogen-activated protein kinases (MAPKs), in various cell types. A recent study showed that the phosphorylation level of JNK in fibroblasts decreases after 72 hours of HG stimulation [13]. By contrast, we found that JNK and ERK phosphorylation levels were constitutively higher in HG-stressed VECs than in LG-treated cells (Fig 4A, 4C, 4E and 4G), while p-JNK level was induced shortly after bFGF treatment and p-ERK level was not changed significantly after bFGF treatment (Fig 4B, 4D, 4F and 4H). However, bFGF treatment for 30 min reduced the HG-stimulated increase in p-JNK and p-ERK levels after 72 hours of HG treatment (Fig 4I, 4J, 4K and 4L). These results suggested that HG continually activates the JNK and ERK. Nevertheless, bFGF regulation on p-JNK level in LG or HG condition was rather a transient process via its signaling pathway. In addition, wound scratch assays demonstrated that ERK and JNK inhibitors (U0126 and sp600125, respectively) inhibited cell migration which was consistent with the identification of the vital role of MAPKs in cell migration (Fig 5A and 5B). Furthermore, U0126 and sp600125 promoted cell migration in HG condition to varying degrees respectively (Fig 5C, 5D and 5E). These results indicated that bFGF promotes cell migration by suppressing HG-induced activation of MAPKs (JNK and ERK).

### 4. JNK and ERK are involved in hyperglycemia-induced ROS production and apoptosis

**Fig 6:**
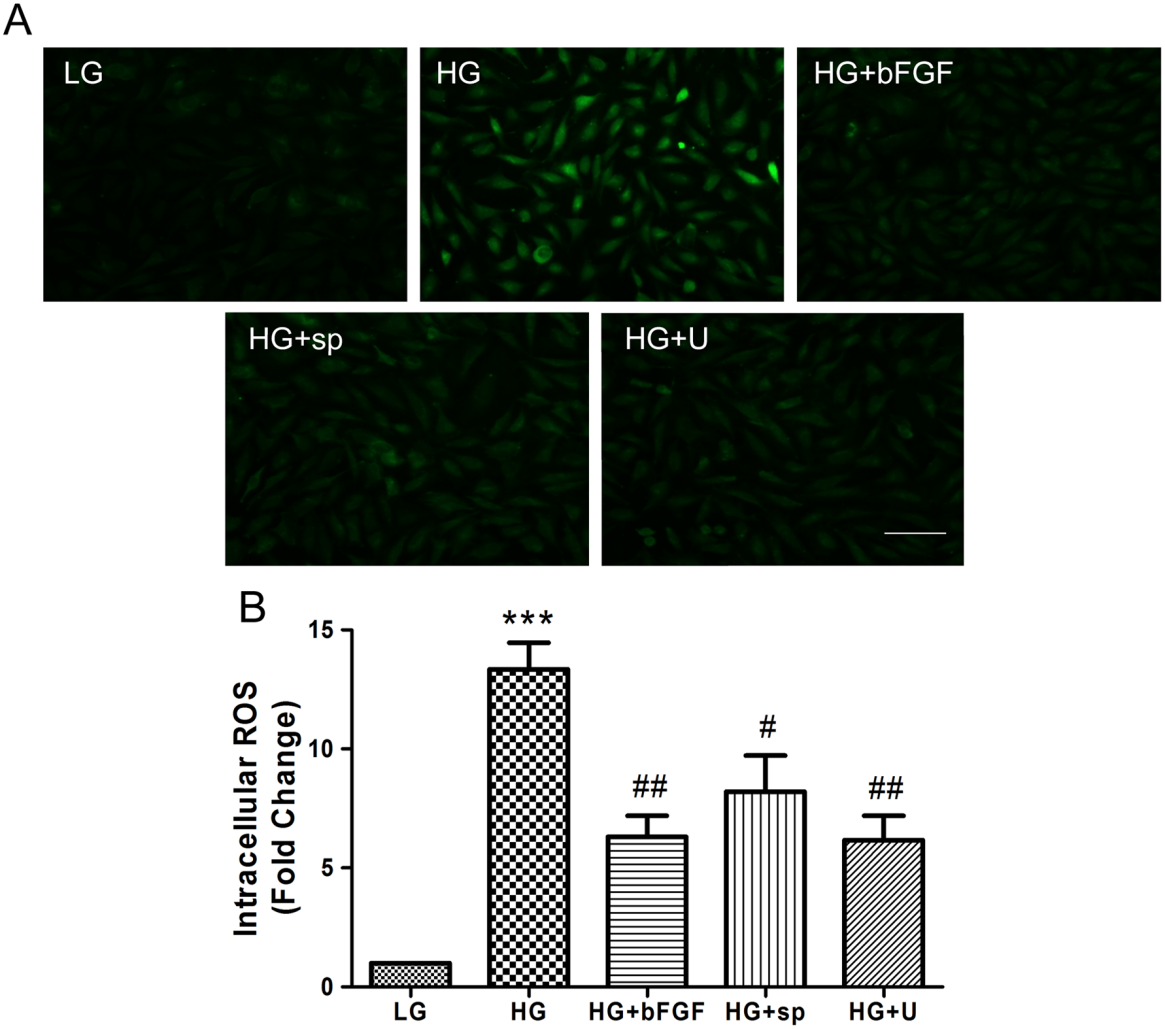
High-glucose increases generation of reactive oxygen species (ROS) in VECs. (A) The probe 2’,7’-dichlorohydrofluorescein (DCFH2-DA) was added to the medium of cells treated with low glucose (LG) or high glucose (HG). HG-treated cells were also treated with 100 ng/mL bFGF, 1 μM sp600125 (sp), or 1 μM U0126 (U). ROS were observed in all cells, especially in high glucose-treated cells. Bar = 100 μm. (B) Fluorescence levels in ten different cells were measured using the Image Pro Plus software. Data represent mean values ± SE of four replicates, relative to the LG group (***P < 0.001) and relative to the HG group (^#^ *P <* 0.05, ^##^ *P <* 0.01).

**Fig 7:**
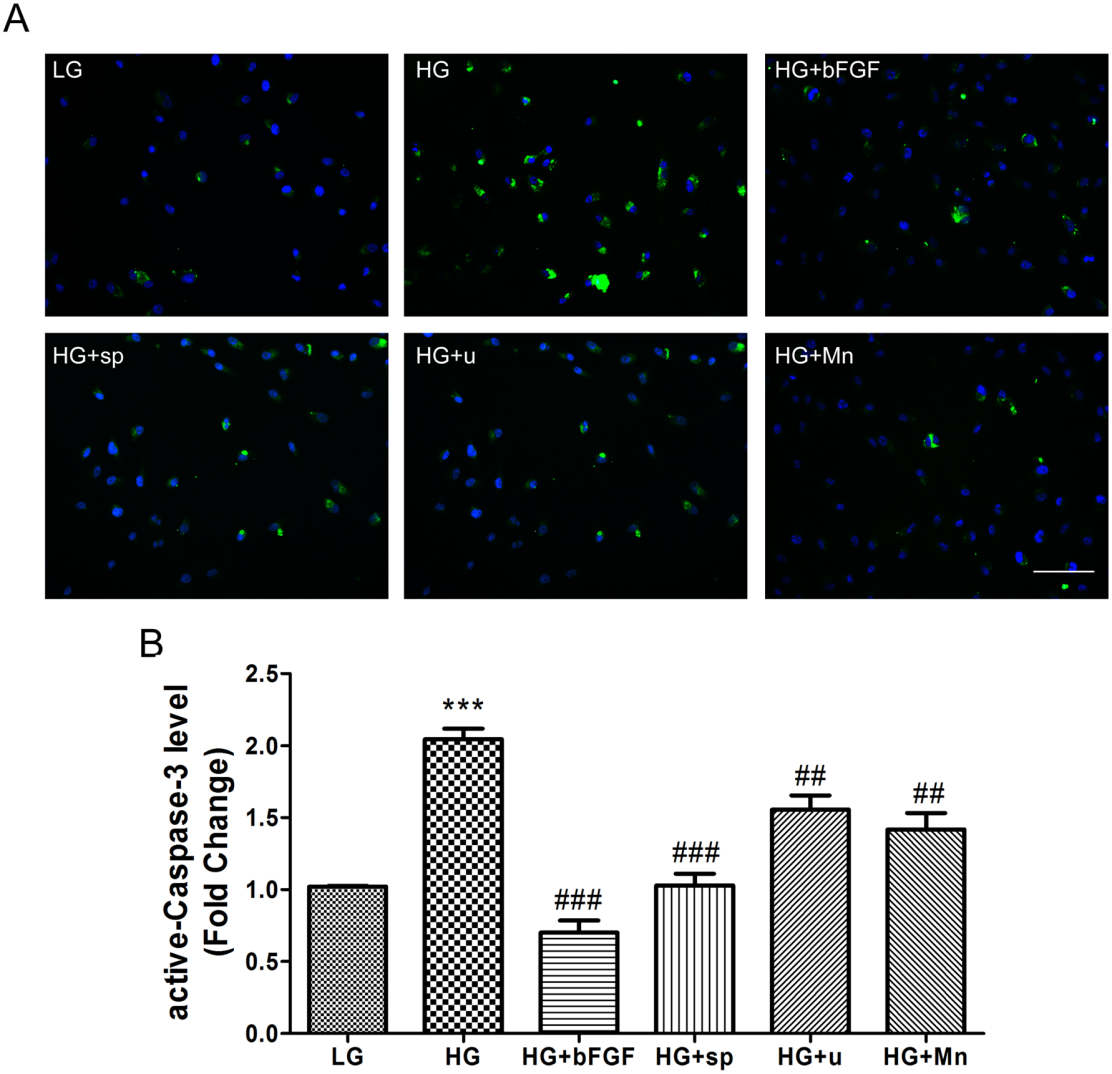
High-glucose induces apoptosis in VEC. (A) Apoptotic responses in VEC were analyzed by detection of active-Caspase-3 immunofluorescence using a-Caspase-3 antibody. Cells were treated with low glucose (LG) or high glucose (HG) for 72 hours before treated with 100 ng/mL bFGF, 1 μM sp600125 (sp), or 1 μM U0126 (U) or 10 μM MnTmPyP for 1 hour. Bar = 100 μm. (B) Fluorescence levels in ten different cells were measured using the Image Pro Plus software. Data represent mean values ± SE of four replicates, relative to the LG group (***P < 0.001) and relative to the HG group (##*P* < 0.01, ###*P* < 0.001). DAPI was used to stain nucleus. Green and blue signals indicate a-Caspase-3 and nucleus respectively.

**Fig 8:**
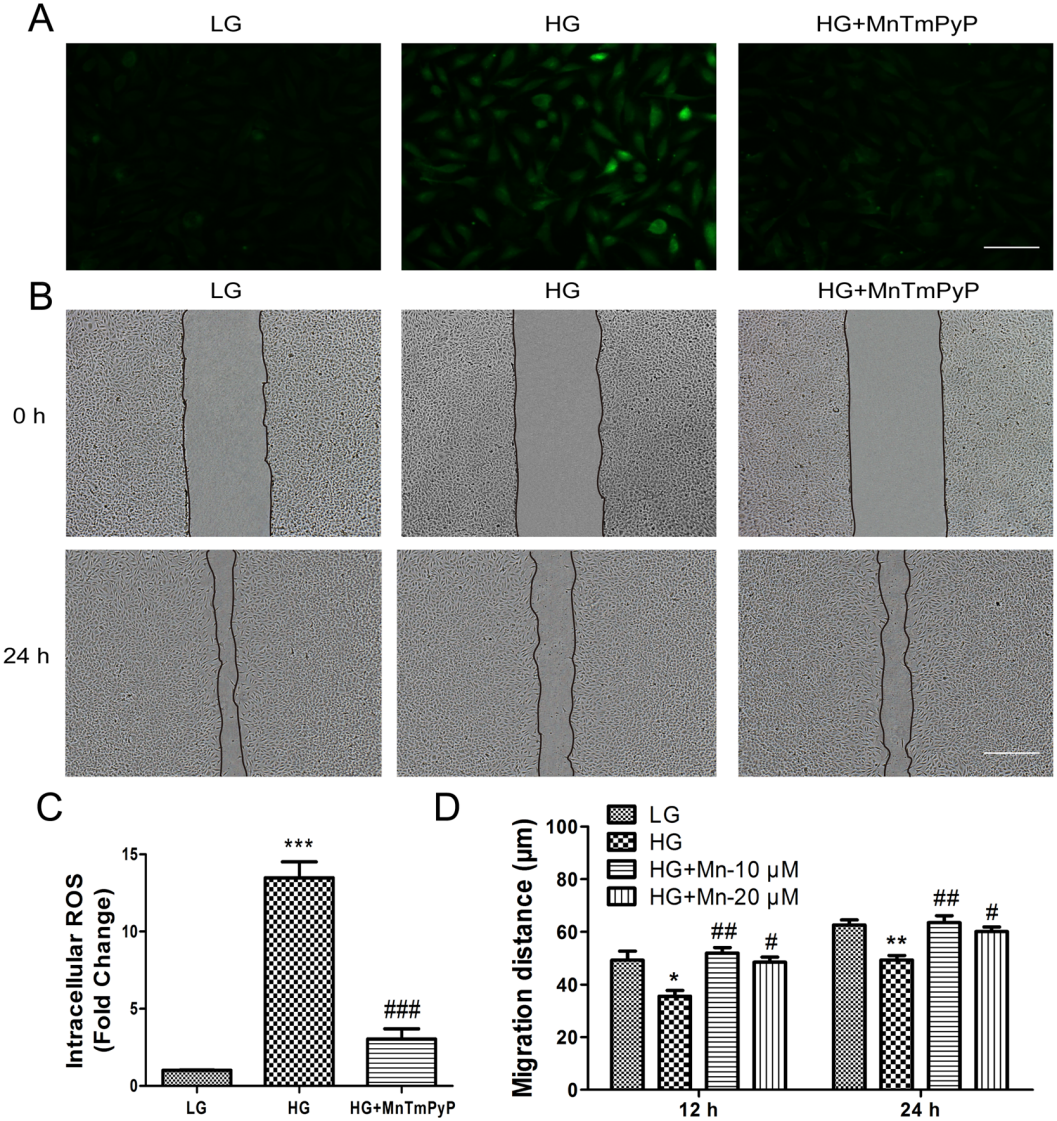
High glucose-induced accumulation of intracellular ROS levels delays VEC migration. (A) Cells were pretreated with high glucose (35 mM) for 72 hours, and then incubated with the ROS scavenger MnTmPyP (10 μM) for 1 hour. Intracellular ROS levels were measured using DCFH-DA dye. Bar = 100 μm. (B) Wound scratch assay was performed to analyze the effects of 10 μM MnTmPyP in human VECs. LG: medium containing 5.5 mM glucose. Bar = 500 μm. All experiments were performed after exposure of cells to 5 mg/mL mitomycin C (to inhibit proliferation) for 1 day. (C) Fluorescence levels in ten different cells were measured using the Image Pro Plus software. Data represent mean values ± SE of four replicates, relative to the LG group (***P < 0.001) and relative to the HG group (###*P <* 0.001). (D) Cell migration distance was determined according to the data shown in (B). Data represent mean values ± SE of ten replicates, relative to the HG group (*P < 0.05, **P < 0.01).

Increased ROS generation is a hallmark of diabetes and active-Caspase-3 level is a representative of apoptosis. To analyze the effect of HG on ROS production and a-Caspase-3 level in VECs, we measured the fluorescence intensity of ROS and a-Caspase-3 in cells cultured for 3 days in LG or HG medium. Cells in HG medium accumulated more ROS and a-Caspase-3 than LG-treated cells (Figs 6A, 6B, 7A and 7B). To better understand the pathways governing hyperglycemia-induced ROS stress and apoptosis, we incubated cells with HG for 72 hours, and then treated them with an inhibitor of JNK (sp600125) or ERK (U0126) for 1 hour. Both compounds markedly attenuated the HG-induced overproduction of ROS and a-Caspase-3 (Figs 6A, 6B, 7A and 7B). In addition, application with a ROS scavenger, MnTmPyP, reduced the ROS production and inhibited cell apoptosis in HG-treated cells (Figs 7A, 7B and 8A). These observations suggested that HG-mediated ROS production and apoptosis in VECs is associated with JNK and ERK signaling.

### 5. HG-induced delay in VEC migration is connected to ROS accumulation

Because HG induced ROS accumulation, we investigated the influence of the ROS level on cell migration. Cells were initially maintained in media containing 1 g/L (5.5 mM) glucose. For HG conditions, cells were transferred to media containing 35 mM glucose for 72 hours, and then treated with the ROS scavenger MnTmPyP (20 μM) for 1 hour. ROS production significantly increased after 72 h with 35 mM glucose, but treatment with MnTmPyP led to an obvious reduction in ROS levels (Fig 8A). Furthermore, reduction of ROS by MnTmPyP treatment reversed the HG-induced delay in cell migration (Fig 8B, 8C and 8D). Taken together, these results indicate that HG-induced ROS production is associated with cell migration processes.

## Discussion

The vascular endothelium plays important roles in maintaining vascular tone and function, in part by synthesizing and releasing vasoactive substances such as nitric oxide. VEC dysfunction contributes to the pathogenesis of vascular diseases in diabetics. In this study, we found the impaired angiogenesis in DM rats and the effects of high glucose (HG) on cell proliferation and migration. Relative to low glucose (LG), HG inhibited both proliferation and migration (Figs 2A and 3A). The mechanisms underlying VEC dysfunction in diabetes mellitus (DM) remain unclear, but one candidate is increased inactivation of endothelium-derived nitric oxide by reactive oxygen species (ROS), [23, 24] which act as signaling intermediates. *In vitro*, high ambient glucose affects endothelial and other vascular cells at the cellular level [25], delays VEC replication [26], and causes excessive cell death [27]. It is reasonable to hypothesize that ROS generation is associated with the HG-induced delay of migration in human VECs, but this relationship has not been previously documented. Moreover, the molecular mechanisms underlying HG-impaired migration require further investigation. The results of this study revealed a statistically significant difference in intracellular ROS levels between HG- and LG-treated VECs, and demonstrated that a reduction in ROS levels restores the ability to migrate.

The c-Jun amino-terminal kinase (JNK) contributes to inflammation, proliferation, and apoptosis [28, 29], and JNK activation promotes cell migration in several systems [30, 31]. Likewise, the extracellular regulated protein kinase (ERK), a mitogen-activated protein kinase, is involved in adhesion turnover in migrating cells [32]. ERK signaling regulates pseudopodium formation, a dynamic process that is necessary for cell migration [33]. In this study, we showed that immoderately activated JNK and ERK in human VECs exposed to HG underwent apoptosis and caused excessive production of ROS, leading to a delay in cell migration. Although cell migration could be restored by inhibition of JNK or ERK inhibitor in HG-stressed cells, but LG incubated cells exposed to either inhibitors exhibited extremely impaired cell migration. (Fig 5A and 5B). Both bFGF and HG induced JNK phosphorylation, but bFGF action on JNK happened at early stage while HG constitutively activated JNK and ERK. These results suggest that moderate activation of MAPKs (JNK, ERK) are vital for cell migration.

bFGF promotes cell migration and proliferation during the wound healing process, e.g., by activating the PI3K–Rac1–JNK pathway to promote fibroblast migration [10]. Our biochemical studies demonstrated that HG activated JNK and ERK phosphorylation, accompanied by delayed migration by human VECs, whereas bFGF reversed the effect of HG on JNK and ERK activation and promoted cell migration. In human foreskin fibroblasts, HG inhibits cell migration by reducing the level of p-JNK [13], implying that differential regulation of JNK in different cell types by HG stress has a similar effect on cell migration.

**Fig 9:**
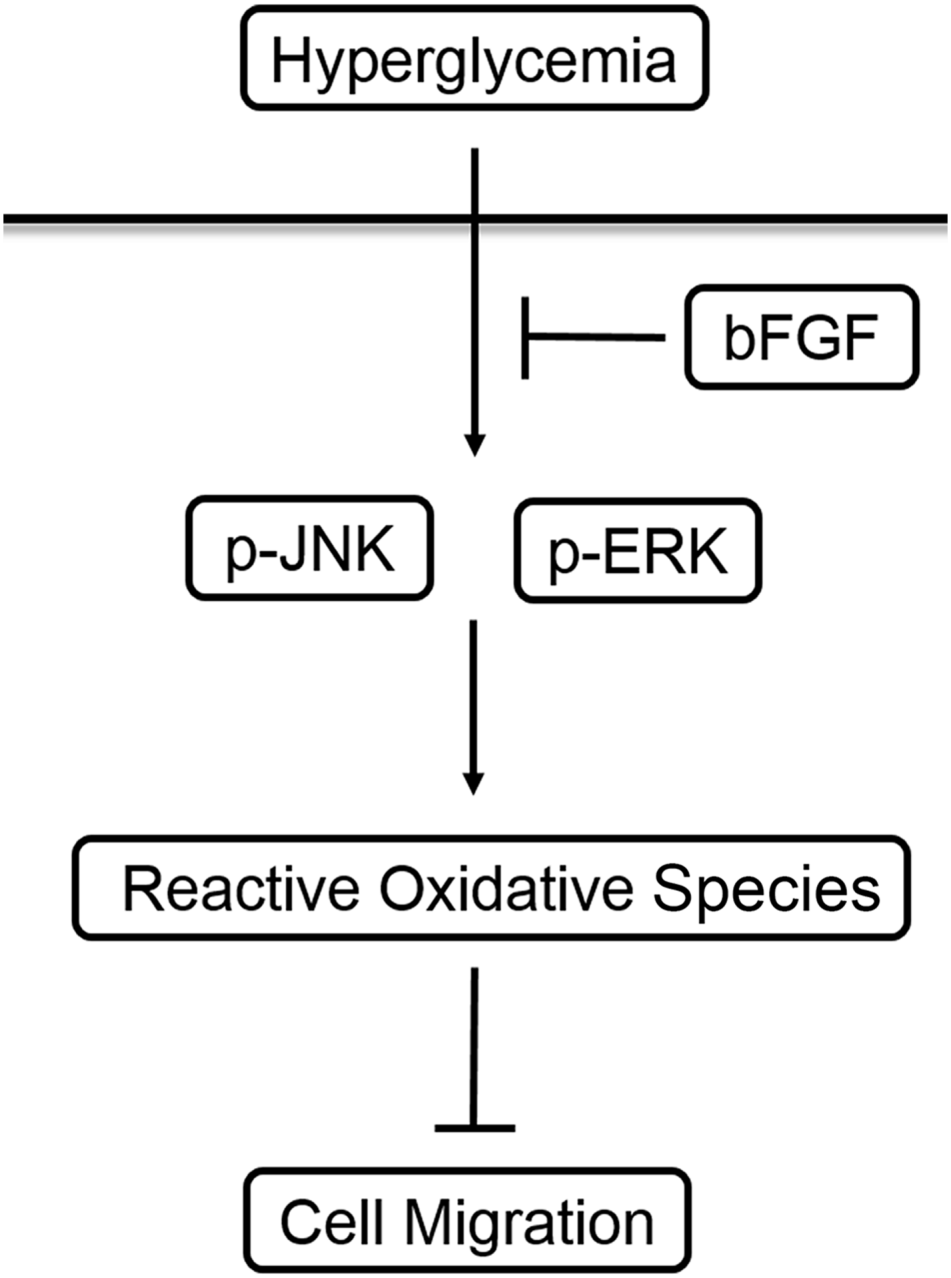
bFGF reverses the high glucose-induced impairment of VEC migration by suppressing ROS accumulation due to activation of JNK and ERK. High glucose was used to simulate hyperglycemia *in vivo*. Hyperglycemia obviously upregulates phosphorylation of JNK and ERK, and the activated forms of these two MAPKs cause accumulation of intracellular ROS, resulting in impairment of cell migration. bFGF treatment of cells incubated in high-glucose media indirectly inhibits the abnormal activation of MAPKs via its signaling pathway, reducing the accumulation of ROS and preventing the delay in cell migration.

In this study, we found that HG activated SAPK/JNK and ERK1/2, consistent with the overproduction of ROS and delay in cell migration (Fig 9). In addition, treatment of VECs with specific inhibitors of JNK and ERK effectively abolished HG-induced ROS production and restored cell migration. Finally, treating VECs with ROS scavenger after HG exposure reduced ROS production and accelerated cell migration. These results suggest that the JNK and ERK1/2 signaling pathways contribute to ROS-mediated impairment of cell migration induced by HG treatment of VECs.
