# Expression of Concern: bFGF-Regulating MAPKs Are Involved in High Glucose-Mediated ROS Production and Delay of Vascular Endothelial Cell Migration

**DOI:** 10.1371/journal.pone.0282770

**Published:** 2023-03-02

**Authors:** The PLOS ONE Editors

After this article [1] was published, concerns were raised about similarities between the wound scratch assays in Fig 5 in article [1] and the wound healing assays in Fig 5 in an *OncoTargets and Therapy* article [2] which was published after article [1] and has since been retracted [3]. In addition to the apparent image duplications noted in [3], the *PLOS ONE* Editors noted concerns about similarities between further wound scratch assays in Figs 3, 5, and 8 in article [1] and the wound healing assays in Figs 2 and 5 in [2].

In response to these concerns, the corresponding author provided all original data underlying Figs 3, 5, and 8 (S1–S2 Files). During editorial follow-up on this underlying data, it was observed that artifacts appeared in some of the underlying data which are not present in article [1] for the corresponding wound scratch assay panels, and that in some cases, there appear to be repeated similarities between different regions in the central section within the same panel in Figs 5 and 8. Specifically:

- The underlying data in S1 File for the 0h, LG and 0h, HG panels in Fig 3A show artifacts that are not present in the corresponding panels in article [1].
- For Fig 5A:
  ○ The underlying data in S1 File for the 0h, LG+sp; 24h, LG; 24h, LG+sp; and 24h, LG+U panels show artifacts that are not present in the corresponding panels in article [1].
  ○ The underlying data in S1 File for the 0h, LG and 0h, LG+U panels show artifacts that are not present in the corresponding panels in article [1], and there appear to be repeated similarities between different regions in the central section within these panels.
- For Fig 5C:
  ○ The underlying data in S1 File for the 0h, HG; 0h, HG+sp; 0h, HG+U; and 24h, HG+U panels show artifacts that are not present in the corresponding panels in article [1].
  ○ The underlying data in S1 File for the 0h, LG panel show artifacts that are not present in the corresponding panel in article [1], and there appear to be repeated similarities between different regions in the central section within this panel.
- The underlying data in S1 File for the 0h, LG; 0h, HG; and 0h, HG+MnTmPyP panels in Fig 8B show artifacts that are not present in the corresponding panels in article [1], and there appear to be repeated similarities between different regions in the central section within these panels.

In response to these concerns, the corresponding author stated that they adjusted the contrast and brightness of some panels in Figs 3A, 5A, 5C, and 8B in article [1] according to a unified standard, and that they edited the central regions of some panels in these figures in order to improve the appearance. The corresponding author also stated that these figures show the migration rate of cells under different conditions, and the migration rate is represented by the distance between the two edges (μm). The remaining underlying data are available from the corresponding author upon request.

In light of the discrepancies between some of the original data underlying Figs 3A, 5A, 5C, and 8B and the panels in [1], and the adjustments to the central regions of some panels in Figs 5A, 5C, and 8B, the *PLOS ONE* Editors issue this Expression of Concern due to concerns about the integrity of data reporting in this article.

## Supporting information

S1 File Underlying data supporting the wound scratch assays in Figs 3A, 5A, 5C, and 8B. (RAR)

S2 File Underlying data supporting the charts in Figs 3B, 5B, 5D-5E, and 8C-8D. (XLSX)
